# American Institute Bulletin

**Published:** 1895-03

**Authors:** 


### AMERICAN INSTITUTE BULLETIN.

American Institute of Homœopathy Bulletin No. 3 is now issued.

The opening session and the promenade concert will take place at the Ocean House on the dates already announced.

The rates at the hotel will be four dollars and five dollars a day. Orders for rooms should be sent immediately to Dr. George B. Peck, of Providence, R. I., who is the Secretary of the Local Committee.

Accommodations may be had also at the Perry House and the Hotel Aquidnick.

An excursion on Narragansett Bay and a clam-bake will take place Saturday, June 22d. An excursion is also projected to Block Island, thirty miles out at sea. Minor excursions of from two to four hours may also be taken to near points.

Many delegates from the West will take the Fall River boat on Wednesday night preceding the opening.

Other interesting details are mentioned in the Bulletin, copies of which, no doubt, all will receive.

